# *Zinc Finger Protein 30* Is a Novel Candidate Gene for Kernel Row Number in Maize

**DOI:** 10.3390/plants14213361

**Published:** 2025-11-03

**Authors:** Yanwei Xiu, Zhaofeng Li, Bin Hou, Yue Zhu, Jiakuan Yan, Feng Teng, Samat Xamxinur, Zhaohong Liu, Naeem Huzaifa, Tudi Anmureguli, Haitao Jia, Zhenyuan Pan

**Affiliations:** 1 Laboratory of Oasis Ecology Agricultural of Xinjiang Production and Construction Corps, Agricultural College, Shihezi University, Shihezi 832003, China; 15910023080@163.com (Y.X.); zhaofengli@shzu.edu.cn (Z.L.); 18699445255@163.com (B.H.); 18290995310@163.com (Y.Z.); 17699932864@163.com (J.Y.); 18449395706@163.com (S.X.); 13313317892@163.com (Z.L.); huzi7922@gmail.com (N.H.); anmuuu@163.com (T.A.); 2 Hubei Key Laboratory of Food Crop Germplasm and Genetic Improvement, Food Crops Institute, Hubei Academy of Agricultural Sciences, Wuhan 430064, China; tengfeng1217@163.com

**Keywords:** maize, kernel row number, quantitative trait locus, transcriptome

## Abstract

Kernel row number (KRN) is a pivotal determinant for yield in maize breeding programs. However, the genetic basis underlying KRN remains largely elusive. To identify candidate genes regulating KRN, a population of 318 BC_4_F_4_ chromosomal segment substitution lines (CSSLs) was developed via backcrossing, with Baimaya (BMY) as the donor parent and B73 as the recurrent parent. Furthermore, a high-density genetic linkage map containing 2859 high-quality single-nucleotide polymorphism (SNP) markers was constructed for quantitative trait locus (QTL) mapping of KRN. Notably, 19 QTLs controlling KRN were detected across three environments and in the Best Linear Unbiased Prediction (BLUP) values; among these, a major-effect QTL (*qKRN4.09-1*) was consistently identified across all three environments and BLUP. Then, the integration of linkage mapping and transcriptome analysis of 5 mm immature ears from near-isogenic lines (NILs) uncovered a candidate gene, *Zm00001eb205550*. This gene exhibited significant downregulation in *qKRN4.09-1^BMY^*, and two missense variants were detected between *qKRN4.09-1^BMY^* and *qKRN4.09-1^B73^*. *Zm00001eb205550* exhibited preferential expression in developing ears. Moreover, the pyramiding of favorable alleles from the five stable QTLs significantly increased KRN in maize. These findings advance our genetic understanding of maize ear development and provide valuable genetic targets for improving KRN in maize breeding.

## 1. Introduction

Maize (*Zea mays* L.), native to South America [1,2], is a globally important cereal crop that serves both as a primary food source and an industrial feedstock. Maize yield primarily depends on three components: ears per unit area, kernels per ear and thousand kernel weight (TKW). Kernels per ear, in turn, can be dissected into two subcomponents: kernels per row and kernel row number (KRN). Teostine, the wild ancestral progenitor of cultivated maize, produces only 2 kernel rows, whereas modern cultivated maize varieties have 8–20 kernel rows [3]. With the aim of addressing accelerating global population growth, the primary goal of maize breeding programs is to enhance yields by optimizing KRN [4,5]. Thus, a comprehensive understanding of the genetic and molecular basis of KRN is essential for developing high-yielding maize varieties.

In maize, KRN is a classic quantitative trait controlled by multiple quantitative trait loci (QTLs) [6]. Mapping QTLs for KRN in maize not only provides molecular markers and candidate regions to decipher the genetic basis of this complex trait, but also lays the groundwork for investigating the molecular mechanisms underlying ear development. Liu et al. detected 33 KRN-associated QTLs across three F_2:3_ populations, of which 21 were consistently identified across multiple environments [7]. Han et al. conducted KRN QTL mapping using a maize F_2:3_ population derived from the cross of V54 and Lian87, identifying 12 KRN-controlling QTLs across four environments, with the phenotypic variance explained (PVE) ranging from 1.40 to 14.95% [8]. Gao et al. developed an immortalized F_2_ (IF_2_) population and identified two stable KRN-associated QTLs across four environments [9]. Nie et al. successfully detected a KRN-associated QTL across F_2_ and F_2:3_ populations [10]. Furthermore, integrating QTL mapping with transcriptome sequencing provides a powerful approach to efficiently identify candidate genes. Three differentially expressed genes (*Zm00001d015377*, *Zm00001d013277* and *Zm00001d015310*) were identified via the transcriptome analysis of 5 mm immature ears of N531, IL_A and IL_B, combined with regional association mapping [11]. By integrating transcriptomic data and genome annotation data, five highly expressed KRN-associated genes (*Zm00001d053756*, *Zm00001d053775*, *Zm00001d053819*, *Zm00001d046783* and *Zm00001d046930*) related to KRN were identified [12]. Three differentially expressed genes (*Zm00001d002996*, *Zm00001d002990* and *Zm00001d002989*) were identified via transcriptome analysis of 3–4 mm immature ears from *qKRN2-1* NILs, combined with QTL mapping [13]. The KRN regulatory gene *krn1* was linked to *ids1/Ts6* via map-based cloning and association mapping. The transcriptome analysis of 1–2 mm immature ears from 133D-GG and 133D-AA further supported the potential role of *krn1* in regulating maize KRN [14]. Despite the identification of several KRN-associated candidate genes, the genetic basis of KRN remains largely elusive.

KRN determination takes place during the early phase of maize ear development [15]. The transition of axillary meristems (AMs) to inflorescence meristems (IMs) at the leaf axils of maize leaves initiates female inflorescence development. Subsequently, the IM proliferates to form spikelet pair meristems (SPMs), which differentiate into two spikelet meristems (SMs). This meristematic patterning establishes the even-numbered kernel row trait in maize. Each SM then undergoes periclinal cell division to produce two floral meristems (FMs), which differentiate into epigynous and hypogynous flowers, respectively. Of these two floral structures, only the epigynous flower undergoes maturation to form a viable seed, while the hypogynous flower aborts during early development. Maize KRN is directly determined by the number of SPMs produced per ear, linking meristem fate to yield trait variation [14,16]. Maize female ear development is further controlled by conserved regulatory pathways: the CLAVATA-WUSCHEL (CLV-WUS) feedback loop [17], microRNA regulation [18], RAMOSA signaling [19], and hormone homeostasis pathways [20]. Key genes in these pathways include *thick tassel dwarf1 (td1)* [21], *fasciated ear2* [3], *fasciated ear3 (fea3)* [16], *corngrass1 (cg1)* [18], *unbranched2 (ub2)*, *unbranched3 (ub3)* [22,23] and *indeterminate spikelet 1*/*tasselseed6* (*ids1/ts6*) [14], all of which modulate meristem proliferation. Notably, the regulatory genes controlling maize KRN have been identified via mutant screens, but these mutants frequently exhibit pleiotropic, unfavorable phenotypes that limit their utility in breeding programs.

Using a BC_4_F_4_ chromosomal segment substitution line (CSSL) population derived from BMY × B73, we detected 19 KRN-associated QTLs across three environments and in BLUP. Of these, five QTLs were consistently detected in two or more environments, while another major-effect QTL (*qKRN4.09-1*) was reproducibly identified across all three environments and in BLUP. Subsequently, integrating linkage mapping with transcriptome profiling of 5 mm immature ears from *qKRN4.09-1* NILs identified a candidate gene: *ZFP30* (annotated as a putative zinc finger protein 30). This gene was previously shown to exhibit vascular tissue-specific expression in developing maize ears via Stereo-seq. Notably, the polymerization of favorable alleles from the five stable QTLs could significantly increase KRN in maize. The research finding will not only enhance our genetic understanding of ear development but also provide genetic targets for the genetic improvement of maize KRN.

## 2. Results

### 2.1. Phenotypic Analysis of KRN in the BC_4_F_4_ Population

**Figure 1:**
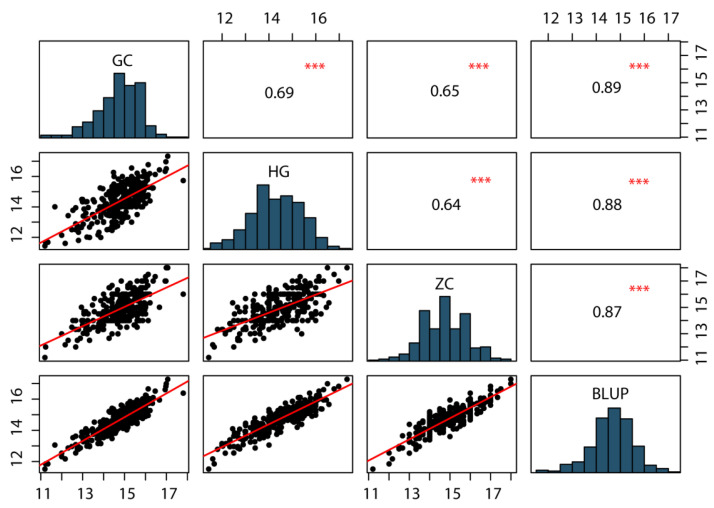
Correlation analysis and frequency distribution of KRN of the BC_4_F_4_ populations in three environments and BLUP. The significance levels between each pair of environments are presented with asterisks. ***, Significant at the 0.001 level.

For phenotypic characterization of KRN, a BC_4_F_4_ population of 318 individuals was developed by crossing the inbred line B73 (recurrent parent) with BMY (donor parent) and was evaluated across three field environments: HG, GC and ZC. The phenotypic data for KRN in the BC_4_F_4_ population across the three environments, along with BLUP, are summarized in Table 1. Notably, KRN exhibited extensive phenotypic variation within the population, ranging from 11.2 to 18 rows per ear, with a coefficient of variation (CV) of 7.0% to 7.54% across the three environments. Furthermore, KRN followed a normal distribution with absolute values of skewness and kurtosis <1 (Table 1, Figure 1), consistent with its being a quantitative trait governed by multiple minor-effect genes. KRN values for the population were significantly positively correlated across the three environments and BLUP values, with correlation coefficients ranging from 0.64 to 0.89 (Figure 1). Moreover, the broad sense heritability (H^2^) of KRN was 87.63% (Table 1), indicating that KRN is phenotypically stable across environments and primarily influenced by genetic variation. Collectively, these findings demonstrate that KRN exhibits extensive genetic variation in the BC_4_F_4_ population and that genetic factors are the major drivers of KRN variation.

### 2.2. Identification of QTLs Associated with KRN in Maize

**Figure 2:**
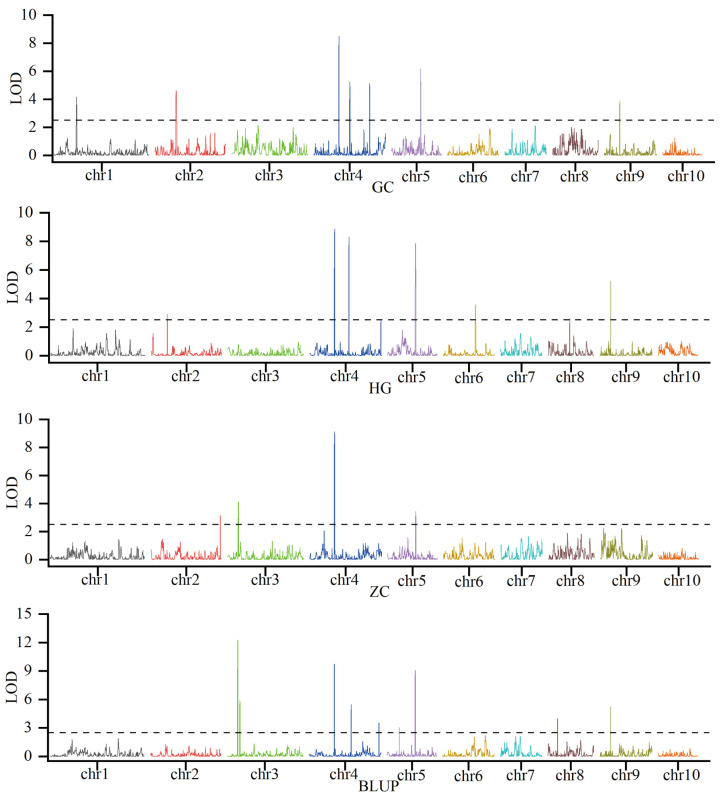
Identification of KRN-associated QTLs in three distinct environments and BLUP. The black dashed line indicates the significance threshold (LOD = 2.5).

To dissect the genetic basis of KRN, linkage mapping was conducted using phenotypic data for KRN and a genotypic panel of 2859 high-quality SNPs which were genotyped using the Maize-SNP10K array. Across three environments and BLUP, which integrated phenotypic variation across trials, 19 KRN-associated QTLs were detected (Figure 2; Table S1). These QTLs were distributed across eight maize chromosomes (1, 2, 3, 4, 5, 6, 8, and 9), and exhibited substantial variation in genetic effect: phenotypic variation explained (PVE) spanned 2.3% to 13.6%, logarithm of odds (LOD) scores ranged from 2.6 to 12.3, and additive effects (Add) varied from −0.64 to 0.38. Critically, QTL reproducibility across environments and BLUP differed markedly, with key stable loci emerging. *qKRN4.09-1* was consistently detected in all three environments and BLUP; *qKRN5.03-1* was identified in two environments and BLUP; *qKRN3.04-1*, *qKRN4.09-2*, and *qKRN9.03-2* were reproducible in one environment and BLUP; and the remaining 14 QTLs were environment-specific (Figure 2; Table S1). Additionally, 11 of these QTLs exhibited negative additive effects, including all the reproducibly detected loci, indicating that the B73 genotype contributes substantially to KRN enhancement in this population. To further resolve the genetic basis of KRN, *qKRN4.09-1* was selected as a key QTL. Its interval spans 231,020,672–233,763,046 bp on chromosome 4, based on the B73 reference genomeV3. For *qKRN4.09-1,* the mean LOD score was 9.05, mean PVE was 10.05%, and the mean Add was −0.50, consistent with this locus being a stable, major-effect QTL (Table S1). Furthermore, the B73-derived allele at this locus is favorable, as it increases KRN by 0.5 rows. Using gene annotation data from the B73 reference genome V5, 71 genes were identified within the *qKRN4.09-1* interval (Table S2).

### 2.3. Analysis of KRN and Inflorescence Meristem of the Near-Isogenic Lines of qKRN4.09-1

**Figure 3:**
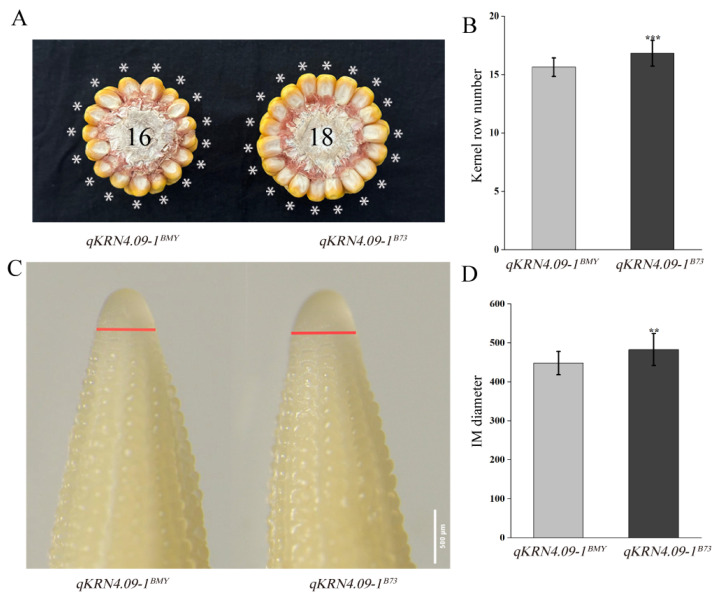
Phenotypic characterization of mature and immature ears of *qKRN4.09-1^BMY^* and *qKRN4.09-1^B73^*. (**A**) Phenotypic photographs of the ears from *qKRN4.09-1^BMY^* and *qKRN4.09-1^B73^*, * indicates KRN of *qKRN4.09-1^BMY^* and *qKRN4.09-1^B73^*. (**B**) Statistical analysis of KRN in mature ears of *qKRN4.09-1^BMY^* and *qKRN4.09-1^B73^*. (**C**) Micrographs of the 5 mm immature ears from *qKRN4.09-1^BMY^* and *qKRN4.09-1^B73^*; the red line indicates the diameter of inflorescence meristem in 5 mm immature ears of *qKRN4.09-1^BMY^* and *qKRN4.09-1^B73^.* (**D**) Statistical analysis of diameter in immature ears of *qKRN4.09-1^BMY^* and *qKRN4.09-1^B73^*. **, Significant at the 0.01 level. ***, Significant at the 0.001 level.

To evaluate the genetic effects of *qKRN4.09-1*, an NIL was developed from the mapping population, designated *qKRN4.09-1^BMY^*, with a genetic background recovery rate of 96.64%. Critically, *qKRN4.09-1^BMY^* shared identical genotypes with the recurrent parent B73 at all other QTLs, eliminating potential confounding genetics effects. The recurrent parent B73 was used as the reference NIL (*qKRN4.09-1^B73^*) for *qKRN4.09-1*. *qKRN4.09-1^BMY^* exhibited a significant reduction in KRN relative to *qKRN4.09-1^B73^* (Figure 3A,B). Previous studies have established that KRN is determined by inflorescence meristem (IM) size [24]. To test whether *qKRN4.09-1* regulates KRN via inflorescence meristem (IM) size, we measured IM diameters from 5 mm immature ears of *qKRN4.09-1^BMY^* and *qKRN4.09-1^B73^*. Consistent with the reduced KRN, *qKRN4.09-1^BMY^* had significantly smaller inflorescence meristem (IM) diameters than *qKRN4.09-1^B73^* (Figure 3C,D).

### 2.4. Transcriptome Analysis of the 5 mm Ears from the Near-Isogenic Lines of qKRN4.09-1

To further dissect the genetic basis of KRN variation between QTLs *qKRN4.09-1^BMY^* and *qKRN4.09-1^B73^*, transcriptome sequencing was performed on the 5 mm immature ears of the NILs. After filtering low quality reads and adapter sequences, 305,153,674 clean reads were generated. Across all samples, more than 97.36% of bases displayed a Phred quality score (Q-score) ≥ Q30. Finally, approximately 94% of reads could be mapped to the B73 reference genome V5 (Table S3).

**Figure 4:**
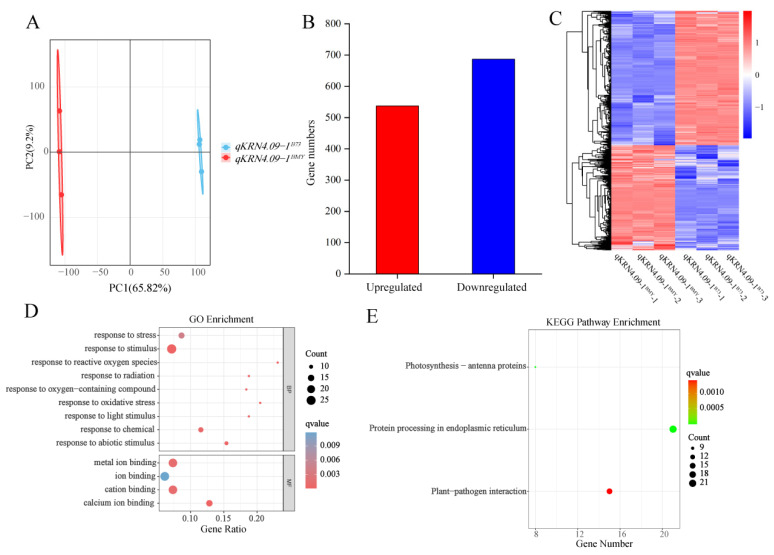
Transcriptomic analysis of near-isogenic lines *qKRN4.09-1^BMY^* and *qKRN4.09-1^B73^*. (**A**) Principal component analysis (PCA) of *qKRN4.09-1^BMY^* and *qKRN4.09-1^B73^*. (**B**) Statistical analysis of the number of up-regulated and down-regulated genes. (**C**) Heatmap clustering of 1224 DEGs based on their expression levels (FPKM). (**D**) GO enrichment of common DEGs. (**E**) KEGG Pathway of common DEGs.

To validate data reliability, principal component analysis (PCA) was performed on the transcriptome datasets. Results revealed robust intra-group reproducibility, with inter-group variation between NILs primarily driven by PC1 (Figure 4A). Using this validated dataset, 1224 differentially expressed genes (DEGs) were identified between the NILs *qKRN4.09-1^BMY^* and *qKRN4.09-1^B73^*, comprising 537 upregulated and 687 downregulated genes (Figure 4B). We next constructed a heatmap depicting the expression patterns of these DEGs across six samples (Figure 4C). These results confirmed high similarity in gene expression patterns between biological replicates of the same genotype, while overall gene expression profiles displayed significant divergence. Next, gene ontology (GO) enrichment analysis was performed on these DEGs (Figure 4D). Applying a significance threshold of q-value < 0.05, 13 significantly enriched GO terms were identified. Within the molecular function category, “ion binding” terms were most highly represented. Additionally, a Kyoto Encyclopedia of Genes and Genomes (KEGG) enrichment analysis was performed on these DEGs to delineate pathways implicated in ear development (Figure 4E). Using a threshold of q-value < 0.05, three enriched KEGG pathways were identified: “Photosynthesis-antenna proteins”, “Protein processing in endoplasmic reticulum” and “Plant-pathogen interaction”. Of these, “Protein processing in endoplasmic reticulum” exhibited the highest significance. Collectively, the analyses identified significant DEGs between *qKRN4.09-1^BMY^* and *qKRN4.09-1^B73^*. Notably, these DEGs were significantly enriched for ion binding-related GO terms and the “Protein processing in endoplasmic reticulum” KEGG pathway; these findings highlight their potential to modulate ear development in maize.

### 2.5. Candidate Gene Prediction in qKRN4.09-1

**Figure 5:**
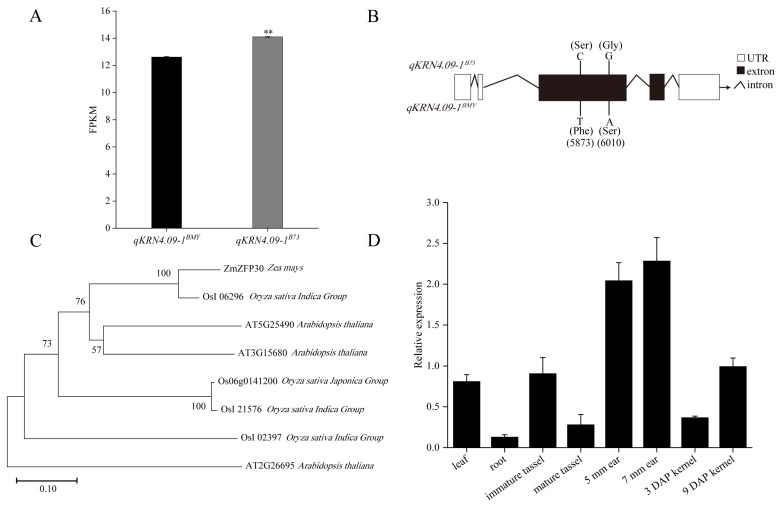
Identification of candidate genes within the *qKRN4.09-1* interval. *(***A**) Fragments per kilobase of transcript per million mapped fragments (FPKM) values of *Zm00001eb205550* (designated as *ZmZFP30*) in *qKRN4.09-1^BMY^* and *qKRN4.09-1^B73^* NILs. **, Significant at the 0.01 level.  (**B**) Sequence differences of *ZmZFP30* between *qKRN4.09-1^BMY^* and *qKRN4.09-1^B73^* NILs. (**C**) Phylogenetic tree analysis of *ZmZFP30* protein among maize, rice and Arabidopsis thaliana. (**D**) RT-qPCR analysis of *ZmZFP30* expression in different tissues. The relative expression levels of *ZmZFP30* in leaves, roots, tassels, ears, and kernels at distinct developmental stages are shown, with three biological replicates per sample. 5 mm ear, the developing ear whose length is about 5 mm. 7 mm ear, the developing ear whose length is about 7 mm. 3 DAP kernel, the kernels at 3 days after pollination. 7 DAP kernel, the kernels at 7 days after pollination.

To identify the candidate gene underlying KRN-associated QTL *qKRN4.09-1*, the expression profiles of genes within the *qKRN4.09-1* interval were analyzed using transcriptome data from NILs. Of the 71 genes in this *qKRN4.09-1* interval, only 44 were detected at the transcriptional level. Critically, only one gene (*Zm00001eb205550*) exhibited differential expression between the NIL pair *qKRN4.09-1^BMY^* and *qKRN4.09-1^B73^*. Specifically, *Zm00001eb205550* was significantly downregulated in *qKRN4.09-1^BMY^* relative to *qKRN4.09-1^B73^* (Figure 5A). Based on functional annotation, this gene encodes a putative zinc finger protein 30 (*ZFP30*) and was designated *ZmZFP30*.

To further validate *ZmZFP30* as a candidate gene underlying *qKRN4.09-1*, the gene was cloned from *qKRN4.09-1^BMY^* and *qKRN4.09-1^B73^* and subjected to targeted resequencing. Targeted resequencing uncovered allelic variation in *ZmZFP30* between *qKRN4.09-1^BMY^* and *qKRN4.09-1^B73^*, consisting of two nonsynonymous mutations within the first exon. A cytosine (C) to thymine (T) transversion at genomic position 5,873 bp and a guanine (G) to adenine (A) substitution at 6,010 bp caused serine (Ser) to phenylalanine (Phe) and glycine (Gly) to serine (Ser) amino acid substitutions, respectively (Figure 5B). Moreover, domain prediction analysis confirmed that *ZmZFP30* encodes three Zn ribbon RanBP2 domains, with the 5,873 bp nonsynonymous mutation mapping to the second functional domain (Figure S1). Collectively, these genetic observation, including allelic variation and nonsynonymous mutations, support *ZmZFP30* as a key candidate gene regulating KRN. Subsequently, BLAST v2.16 analysis of the *ZmZFP30* protein sequence was performed, identifying orthologues in Arabidopsis and rice. *ZmZFP30* exhibited the highest sequence identity with OsI_06296 (Figure 5C). However, the relevant functions of this protein remain uncharacterized.

To investigate the function of *ZmZFP30* during maize growth and development, real-time quantitative PCR (RT-qPCR) analysis was performed on diverse tissues and developmental stages, including leaves, roots, immature tassels, mature tassels, 5 mm ears, 7 mm ears, and kernels at 3 and 9 days after pollination (DAP). *ZmZFP30* exhibited significant tissue- and stage-specific patterns of relative expression. Notably, *ZmZFP30* was most highly expressed in the developing ears (5 mm and 7 mm), with substantially lower expression in other tissues, particularly roots (Figure 5D). This expression pattern supports a pivotal role for *ZmZFP30* in regulating maize ear development.

### 2.6. Analysis of the Favorable Allele Frequency Distribution and Aggregation Effect

**Figure 6:**
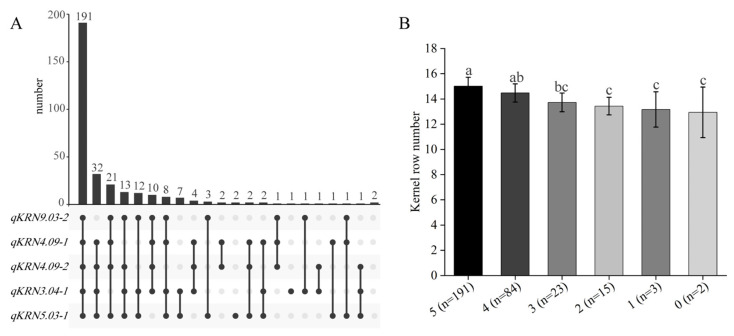
Screening of favorable allele-polymerized materials and KRN comparison among materials with different numbers of favorable alleles. (**A**) UpSet Plot showing the distribution of materials carrying five target QTLs. (**B**) Comparison of KRN of materials with different numbers of favorable alleles. The same lowercase letters represent no significant difference (*p* > 0.05), while distinct lowercase letters indicate significant difference (*p* < 0.05).

Of the 19 identified QTLs, *qKRN3.04-1*, *qKRN4.09-1*, *qKRN4.09-2*, *qKRN5.03-1* and *qKRN9.03-2* were detected across multiple environments and in BLUP, and were thus classified as major QTLs. To identify germplasm resources carrying multiple favorable alleles of these major QTLs, the aggregation of superior alleles was analyzed in the BC_4_F_4_ population. Consequently, 191 germplasm accessions carrying all five favorable QTL alleles were identified, exhibiting significantly higher KRN relative to other genotypes (Figure 6A,B). Further, the KRN across germplasm accessions carrying different numbers of favorable QTLs was compared. Relative to germplasm accessions with four or five favorable QTLs, those carrying zero, one, two, or three favorable alleles exhibited significantly lower KRN. Additionally, pyramiding of all five favorable alleles increased KRN by 2.1 rows. Collectively, these results demonstrate that pyramiding favorable alleles significantly increases maize KRN, providing a theoretical foundation for polygenic breeding in maize.

## 3. Discussion

### 3.1. The Polymerization of Favorable Alleles Can Significantly Increase KRN in Maize

KRN is an important trait related to yield, and enhancing KRN is a key objective in crop breeding programs [25]. However, the genetic basis underlying KRN variation is complex, involving multiple genes and regulatory pathways. QTL mapping is a widely utilized approach for elucidating the genetic architecture of phenotypic variation and has been extensively applied to dissect key agronomic traits in maize [26,27,28,29]. Recent studies have identified numerous KNR-related QTLs [8,10,14,24,30,31]. However, individual QTLs confer KRN increases of less than one row. For instance, the favorable allele *qkrnw4* from inbred line B73 confers KRN by 0.73-0.92 rows [10]. Compared with *qKRN8^lian87^*, *qKRN8^V54^* could increase KRN by 0.43-0.53 rows [8]. The favorable allele of *qKRN5b* could significantly increase KRN by 0.63-0.81 rows [30]. In this study, we identified 19 KRN-associated QTLs using the high-density genetic maps, of which 5 QTLs were detected in at least one environment, and 1 QTL (*qKRN4.09-1*) was mapped in three environments and the BLUP, explaining 7.9% to 13.6% of the phenotypic variation across environments. More importantly, the polymerization of five favorable alleles could increase KRN by 2.1 rows. Meanwhile, all five favorable alleles were derived from B73, demonstrating its value as a crucial germplasm donor for KNR genetic improvement in maize. These results demonstrate the feasibility of molecular pyramiding breeding for maize KRN improvement.

In addition, previous studies have identified numerous QTLs associated with maize KRN, which are distributed across all ten chromosomes. For example, Li et al. integrated 324 QTLs related to KRN from published databases [6]. Zhang et al. summarized 73 QTLs for KRN by integrating public data [32]. Among the five favorable QTLs in the study, *qKRN4.09-2* and *qKRN5.03-1* have been identified in different populations [12,33]. The remaining three QTLs, *qKRN3.04-1*, *qKRN4.09-1*, and *qKRN9.03-2*, have not been detected in previous studies, indicating their novelty. Thus, the integration of previous studies and the three novel genetic loci identified in this study provides new targets for gene mining and molecular breeding of maize.

### 3.2. The Integration of Linkage Mapping and Transcriptome Analysis Can Accelerate the Identification of Genes Related to Complex Traits

QTL fine-mapping procedures, which require the generation of sufficient recombination events, remain time-consuming and laborious. In order to overcome the shortcomings of traditional QTL cloning, the integration of QTL mapping and transcriptome analysis has been widely adopted in gene identification studies. For example, in maize, 10 kernel size-related QTLs in F_2:3_ populations were determined, and by combining these with transcriptome analysis, 11 DEGs were identified [34]. In rice, a major QTL (*qGN4-1)* was mapped in a recombinant inbred line population, and eight DEGs were identified via transcriptome sequencing [35]. In wheat, 10 stable QTLs associated with thousand grain weight were identified in the RIL population. Through the joint analysis of transcriptomic and genomic data, 134 putative candidate genes were mined [36]. In this study, to identify the candidate genes regulating KRN within the *qKRN4.09-1* interval, transcriptome sequencing was performed on 5 mm immature ears of NILs. This developmental stage is critical for KRN differentiation [10,37]. Notably, genes regulating KRN typically exhibit tissue-specific expression at this critical stage. Additionally, NILs are ideal materials for comparative transcriptome analysis due to their shared genetic background. Ultimately, one DEG, *Zm00001eb205550*, was identified within the *qKRN4.09-1* interval: this gene exhibited higher expression in NIL^B73^ with higher KRN. Gene function annotation revealed that *Zm00001eb205550* encodes a putative zinc-finger protein. In summary, the integrated strategy of QTL mapping and RNA-seq facilitated the rapid identification of candidate genes within the target genomic interval. Notably, the selection of appropriate materials, specific tissues and critical developmental stages was essential to this process.

### 3.3. ZmZFP30 Is a Key Candidate Gene Regulating KRN Development in Maize

Zinc-finger protein is a type of transcription factor that forms a finger-like structure through stabilization by Zn^2+^, which is involved in the regulation of plant growth, development and stress adaptation [38,39]. In rice, *LRG1* encoded a ZOS4-06-C2H2 zinc-finger protein and influenced grain size by regulating cell division and expansion in the spikelet hull [40]. *NSG1* encoded a member of the C2H2 zinc finger protein family and played a pivotal role in maintaining organ identities in the spikelet by repressing the expression of LHS1, DL, and MFO1 [41]. In Arabidopsis, *SGR5* encoded a zinc finger protein, and might be involved in an early event in inflorescence stems [42]. *ZIM* encoded a zinc finger protein, and was a putative transcription factor involved in inflorescence and flower development [43]. In this study, a zinc-finger protein, *ZmZFP30*, was identified as being associated with KRN differentiation in maize. Furthermore, *ZmZFP30* contains three structural domains, and resequencing analysis identified a non-synonymous mutation within its second functional domain. Interestingly, prior studies using Stereo-seq technology demonstrated that *ZmZFP30* exhibited specific expression in the vascular tissues of developing maize ears [44]. Consistent with this, our tissue-specific expression analysis revealed higher *ZmZFP30* expression in the 5 mm immature ears. Collectively, these findings support the designation of *ZmZFP30* as a key candidate gene regulating KRN development in maize.

Further, GO enrichment analysis revealed that genes associated with calcium ion binding were significantly enriched in the DEGs. As second messengers, calcium ions are widely involved in the maintenance and differentiation of plant meristems. During maize ear development, calcium ion signaling is hypothesized to regulate the expression of genes associated with meristem activity. Chou et al. demonstrated that Ca^2+^ signaling regulates the expression of *WUS* and *FAF2*, thereby restricting stem cell proliferation and maintaining the size of the meristem [45]. In this study, the enrichment of the calcium ion binding-related genes among downregulated DEGs is hypothesized to have affected the cell division and differentiation rates of inflorescence meristems, ultimately resulting in reduced KRN. Additionally, among the significantly enriched KEGG pathways of the DEGs from the 5 mm ears transcriptome, the “protein processing in the endoplasmic reticulum” pathway contained a relatively substantial number of DEGs. This suggests that *ZmZFP30* is likely involved in regulating the expression of genes associated with endoplasmic reticulum protein processing. Dong et al. revealed that the zinc finger protein *ZmDi19-7* coordinates auxin-mediated cell expansion and the expression of genes involved in endoplasmic reticulum protein processing [46]. However, the molecular mechanism by which *ZmZFP30* regulates calcium signaling pathways and protein processing in the endoplasmic reticulum pathway remains to be further investigated.

## 4. Materials and Methods

### 4.1. Plant Materials and Field Trials

A CSSL population was used, composed of the 318 BC_4_F_4_ population, comprising 318 BC_4_F_4_ progenies developed via backcrossing using Baimaya (BMY) as the donor parent and B73 as the recurrent parent [47]. BMY, an excellent local variety, was collected in Zhushan County, Hubei Province, China. The BC_4_F_4_ population was phenotypically evaluated using a completely randomized design (CRD) with two biological replicates across three geographically distinct environments: Gucheng (GC, 111°07′ E, 32°11′ N), Huanggang (HG, 115°16′ E, 30°38′ N) and Zengcheng (ZC, 113°46′ E, 23°21′ N). Prior to sowing, all plots were uniformly tilled to a depth of 20 cm using a rotary tiller, and base fertilizer was applied at a consistent rate of 300 kg ha^−1^. The double-row plot was adopted. The length of each row was 3 m, the row spacing was 0.70 m, the plant spacing was 0.25 m, and 24 plants were planted in each plot. The phenotypic data employed for QTL detection in the BC_4_F_4_ population all relied on the average values of two repetitions within a single environment. Standard local field management practices were uniformly implemented across all environments.

### 4.2. Collection and Analysis of KRN of the BC_4_F_4_ Population

KRN was determined by counting kernel rows at the mid-section of each ear. At least eight representative ears per line were sampled in each replicate. Phenotypic data were analyzed using SPSS 25. Basic statistical parameters including mean, standard deviation, coefficient of variation, skewness, and kurtosis were calculated. Correlation analyses and frequency distribution histograms were generated using R version 4.4.2. Then, the R package lme4 was employed to estimate broad-sense heritability (H^2^) and Best Linear Unbiased Prediction (BLUP). The H^2^ was estimated using the following formula:

$$H^{2}=\frac{Vg}{Vg+Vge/n+Ve/rn}$$

where Vg denotes the genetic variance, Vge denotes genotype by environment interaction (G × E interaction), Ve denotes the residual variance, n denotes the number of environments and r denotes the number of replications per environment [48].

### 4.3. Construction of a Genetic Linkage Map

Methods of linkage map construction have been described previously [47]. Genomic DNA was isolated from fresh leaf tissues using the cetyl trimethyl ammonium bromide (CTAB) method [49]. DNA quality and quantity were assessed using 1% agarose gel electrophoresis and a NanoDrop 2000 spectrophotometer (Thermo Fisher Scientific, Waltham, MA, USA). DNA samples were considered qualified if they had an OD260/280 ratio of 1.8 and 2.0 and exhibited a single, sharp, and bright main band. Genotyping was performed on the qualified DNA samples from 318 BC_4_F_4_ individuals using the 10K maize chip (MolBreeding, Shijiazhuang, China), yielding a total of 9843 SNP markers. SNP markers were filtered according to the following quality control criteria: minor allele frequencies (MAF) ≥ 0.05, missing call rate < 0.10, and heterozygous rate < 0.50. This filtering yielded 2859 high-quality SNP markers. A genetic linkage map was constructed using JoinMap v4.0 software, with an average inter-marker genetic distance of 1.56 centiMorgan (cM) and a total map length of 4467.48 cM.

### 4.4. QTL Analysis

QTL mapping for maize KRN was conducted across three distinct environments and the BLUP dataset using the inclusive composite interval mapping (ICIM-ADD) method in the QTL IciMapping v4.2 software, with default parameters (PIN = 0.001; walking step = 1.0 cM) [50]. QTLs with a logarithm of odds (LOD) score > 2.5 were defined as major-effect QTLs [51,52]. If the intervals of detected QTLs overlapped across different environments, they were considered to represent the same QTL and were assigned a common designation. All detected QTLs were named starting with ‘q’, followed successively by the abbreviated form of the trait, chromosome number, and the bin marker on the same chromosome. For example, *qKRN4.09-1* indicates the tenth bin marker located on chromosome 4 within the BC_4_F_4_ population responsible for regulating the kernel row number.

### 4.5. RNA Isolation and Transcriptome Analysis

In this study, 5 mm immature ears of near-isogenic lines *qKRN4.09-1^BMY^* and *qKRN4.09-1^B73^* were collected at the 11-leaf stage. Each experimental sample consisted of three biological replicates. The EASYspin Plus Plant RNA Kit (Aidlab Biotech, Beijing, China) was used for RNA extraction. Library construction and sequencing were performed by Wuhan Xinbolai Biotechnology Co., Ltd. (Wuhan, China) using an Illumina HiSeq platform (Illumina, San Diego, CA, USA). Following quality control of the sequencing data, the filtered reads were aligned to the B73 reference genome (B73 RefGen_v5). Raw sequencing counts were converted to FPKM using the R language. Cluster analysis and principal component analysis (PCA) were performed on diverse samples. Then, differentially expressed genes (DEGs) were screened using the thresholds of |log2 Fold Change (FC)| ≥ 1 and Padj < 0.05. Enrichment analysis of the DEGs was conducted in R to elucidate their potential functions. If both the *p*-value and Q-value were less than 0.05, the results of Gene Ontology (GO) and Kyoto Encyclopedia of Genes and Genomes (KEGG) analyses were considered statistically significant.

### 4.6. RNA Extraction and Quantitative Real-Time RT-PCR (qRT-PCR)

Leaves, roots, immature tassels, mature tassels, 5 mm ears, 7 mm ears, 3 DAP kernels and 9 DAP kernels were collected from B73. Total RNA extraction from each sample was performed with the EASYspin Plus Plant RNA Kit (Aidlab Biotech, Beijing, China), after which reverse transcription (RT) was implemented using the EasyScript One-Step gDNA Removal and cDNA Synthesis SuperMix Kit (TransGen Biotech, Dalian, China). Primer3Plus was used to design gene-specific primers for the target gene. RT-qPCR analysis was performed using SYBR Green qPCR Super Mix (TransGen Biotech, Beijing, China) on a C1000 Touch Thermal Cycler (Bio-Rad, Hercules, CA, USA). The 2^−ΔΔC^ method was employed to evaluate the relative expression levels of the target genes, using actin as the internal reference gene [53]. Primer sequences for target gene amplification are provided in Table S4.

### 4.7. Resequencing and Sequence Analysis of Candidate Genes

Samples were collected from the leaves of *qKRN4.09-1^BMY^* and *qKRN4.09-1^B73^* plants grown in the experimental field in Shihezi, with DNA extracted using the FastPure Plant DNA Isolation Mini Kit (Vazyme, Nanjing, China). Reference sequences corresponding to the candidate genes were acquired from MaizeGDB (https://maizegdb.org/), and primers were designed using Primer 3 Plus software. The DNA of *qKRN4.09-1^BMY^* and *qKRN4.09-1^B73^* was used as templates for PCR amplification with specific primers (Table S4). The PCR-amplified products were cloned into a T-vector and subjected to sequencing.

### 4.8. Analysis of Candidate Gene Phylogenetic Tree

Sequences of maize zinc finger proteins were retrieved from MaizeGDB (https://maizegdb.org/). Homologous proteins from Arabidopsis and Oryza Sativa were retrieved from the National Center for Biotechnology Information (NCBI) database (https://www.ncbi.nlm.nih.gov/). A phylogenetic tree of the zinc finger protein family was constructed using MEGA11 software (https://www.megasoftware.net/).

### 4.9. Screening of Favorable Alleles and Analysis of Their Polymerization Effects

Favorable alleles corresponding to the five major QTLs identified in this study were first screened based on QTL mapping results. The number of favorable alleles carried by each individual in the BC_4_F_4_ population was then counted. Finally, the KRN phenotypic values of individuals with different numbers of favorable alleles were compared to evaluate the pyramiding effect of these alleles.

## 5. Conclusions

In this study, 19 KRN-associated QTLs were identified using a BC_4_F_4_ population, with phenotyping conducted across three environments and the BLUP. Among these QTLs, five were stable and consistently detected in two or more environments, and one major-effect QTL (*qKRN4.09-1*) was consistently identified across all three environments and the BLUP. Furthermore, integrating linkage mapping and transcriptome profiling identified a candidate gene (*Zm00001eb205550)*, annotated as putative zinc finger protein 30 and designated *ZmZFP30*. Moreover, *ZmZFP30* exhibited tissue-specific expression in developing maize ears. More importantly, pyramiding of favorable alleles from these five stable QTLs significantly increased maize KRN. Collectively, these findings will not only advance our genetic understanding of maize ear development but also provide valuable genetic targets and a feasible strategy for KRN improvement in maize breeding.

## Figures and Tables

**Table 1:** Statistical analysis of KRN for the BC_4_F_4_ populations in different environments.

Trait	Env.	Mean ± SD	Range	CV (%)	Kurtosis	Skewness	Heritability (%)
KRN	GC	14.78 ± 1.04	11.20~17.80	7.00	0.99	−0.71	87.63
	HG	14.38 ± 1.06	11.43~17.33	7.40	−0.07	−0.17	
	ZC	14.89 ± 1.12	11.20~18.00	7.54	0.12	0.01	
	BLUP	14.67 ± 0.88	11.52~17.27	6.03	0.86	−0.41	

Env., environment. GC, Gucheng. HG, Huanggang. ZC, Zengcheng. CV, Coefficient of Variation. BLUP, Best Linear Unbiased Prediction.

## Data Availability

The raw RNA-seq data from this study have been deposited in China National Center for Bioinformation/Beijing Institute of Genomics, Chinese Academy of Sciences (https://ngdc.cncb.ac.cn) with the accession number CRA032522.

## Supplementary Materials

The following supporting information can be downloaded at: https://www.mdpi.com/article/10.3390/plants14213361/s1, Table S1: Putative QTL identified of KRN for the BC_4_F_4_ populations in different environments; Table S2: List of genes and functional annotations within the *qKRN4.09-1* interval; Table S3: RNA-seq reads of 5 mm immature ear mapped to the maize B73 RefGen_V5 genome; Table S4: Gene-specific primers used in this study; Figure S1: The protein structure of ZmZFP30. The blue column in the Pfam represents the functional domain.
