# Supplementary material for: Zinc Finger Protein 30 Is a Novel Candidate Gene for Kernel Row Number in Maize
Source: Plants (Basel). 2025 Nov 3;14(21):3361. doi: 10.3390/plants14213361 (PMC12608952; doi:10.3390/plants14213361)
Supplement: Supplementary file 1 [file plants-14-03361-s001.zip › plants-3910901-supplementary.pdf]

Table S1 Putative QTL identified of KRN for the BC_4F_4 populations in different environments

QTL	Env.[a]	Chr.[b]	QTL interval	LOD	PVE (%)[c]	Add[d]
qKRN1.06	GC	1	180,540,455-187,127,899bp	4.2	4.8	-0.36
qKRN2.02	HG	2	9,450,541-9,945,149bp	2.9	2.7	0.27
qKRN2.03	GC	2	19,373,048-20,542,050bp	4.6	5.3	0.32
qKRN2.08	ZC	2	220,480,576-220,605,824bp	3.1	4.2	0.22
qKRN3.04-1	ZC	3	13,471,669-19,266,199bp	4.1	5.8	-0.43
qKRN3.04-1	BLUP	3	13,471,669-19,266,199bp	12.3	10.2	-0.50
qKRN3.04-2	BLUP	3	18,013,572-21,762,820bp	5.9	5.0	0.29
qKRN4.04	GC	4	27,048,673-28,950,125bp	5.1	5.8	-0.30
qKRN4.08-1	BLUP	4	181,909,646-183,119,721bp	3.4	2.9	0.15
qKRN4.08-2	GC	4	186,046,646-188,668,664bp	5.2	6.1	-0.41
qKRN4.09-1	GC	4	233,751,037-233,763,046bp	8.5	9.9	-0.45
qKRN4.09-1	HG	4	231,020,672-233,763,046bp	8.9	8.8	-0.52
qKRN4.09-1	ZC	4	231,020,672-233,763,046bp	9.1	13.6	-0.64
qKRN4.09-1	BLUP	4	233,751,037-233,763,046bp	9.7	7.9	-0.40
qKRN4.09-2	HG	4	200,330,535-221,759,123bp	8.3	8.1	-0.48
qKRN4.09-2	BLUP	4	208,817,129-209,429,698bp	5.5	4.2	-0.37
qKRN5.03-1	HG	5	15,976,063-25,824,467bp	7.9	7.7	-0.63
qKRN5.03-1	ZC	5	15,976,063-25,824,467bp	3.4	4.6	-0.46
qKRN5.03-1	BLUP	5	15,976,063-25,824,467bp	9.1	8.0	-0.54
qKRN5.03-2	GC	5	14,774,874-17,912,507bp	6.2	7.5	-0.47
qKRN5.04	BLUP	5	96,568,393-96,568,607bp	3.0	2.3	-0.19
qKRN6.05	HG	6	132,164,713-134,019,348bp	3.6	3.4	0.36
qKRN8.03	HG	8	76,687,543-93,949,576bp	2.6	2.6	0.25
qKRN8.06	BLUP	8	155,182,646-153,247,707bp	4.0	3.0	0.38
qKRN9.03-1	GC	9	79,344,468-88,296,618bp	3.8	4.5	-0.30
qKRN9.03-2	HG	9	89,249,537-90,399,820bp	5.2	5.0	-0.31
qKRN9.03-2	BLUP	9	89,249,537-90,399,820bp	5.2	4.0	-0.24

[a]Env, environment, Gucheng(GC), Huanggang(HG), Zengcheng(ZC), Best Linear Unbiased Prediction (BLUP). [b]Chr, chromosome. [c]PVE, phenotypic variance explained. [d]Add, positive and negative values indicated additive effects by the alleles of B73 and BMY, respectively

Table S2 List of genes and functional annotations within the *qKRN4.09-1* interval

Gene ID (v5)	Description
Zm00001eb 205230	Serine/threonine-protein kinase
Zm00001eb 205240	Protein kinase domain-containing protein; Protein kinase protein with adenine nucleotide alpha hydrolase-like domain
Zm00001eb 205250	Serine/threonine-protein phosphatase
Zm00001eb 205260	Protein CLP1 homolog
Zm00001eb 205270	Uncharacterized protein
Zm00001eb 205280	Ethylene receptor; GAF domain-containing protein
Zm00001eb 205290	Histone deacetylase; Histone deacetylase domain-containing protein; histone deacetylase
Zm00001eb 205300	Uncharacterized protein
Zm00001eb 205310	Pectate lyase
Zm00001eb 205320	unknown
Zm00001eb 205330	Uncharacterized protein
Zm00001eb 205340	Uncharacterized protein; Embryo surrounding factor 1 brassicaceae domain-containing protein
Zm00001eb 205350	ABC transporter A family member 7; ABC transporter domain-containing protein
Zm00001eb 205360	ABC transporter A family member 7
Zm00001eb 205370	Uncharacterized protein
Zm00001eb 205380	Syntaxin-123
Zm00001eb 205390	Uncharacterized protein
Zm00001eb 205400	Secreted protein
Zm00001eb 205410	Uncharacterized protein
Zm00001eb 205420	unknown
Zm00001eb 205430	Coatomer subunit beta'
Zm00001eb 205440	unknown
Zm00001eb 205450	Putative ARF GTPase-activating domain family protein isoform 1
Zm00001eb 205460	unknown
Zm00001eb 205470	Putative SPOC domain / Transcription elongation factor S-II protein
Zm00001eb 205480	ABC transporter G family member 40
Zm00001eb 205490	RNA-binding (RRM/RBD/RNP motifs) family protein; RRM domain-containing protein
Zm00001eb 205500	AAA+ ATPase domain-containing protein; 26S protease regulatory subunit 8
Zm00001eb 205510	Cytochrome P450 734A1; Uncharacterized protein
Zm00001eb 205520	unknown
Zm00001eb 205530	Phenylalanine ammonia-lyase
Zm00001eb 205540	Peptidyl-prolyl cis-trans isomerase CYP95; PPIase cyclophilin-type domain-containing protein
Zm00001eb 205550	RNA-binding protein cabeza; Putative zinc finger protein30; RanBP2-type domain-containing protein
Zm00001eb 205560	NB-ARC domain-containing protein; Apoptotic protease-activating factor 1
Zm00001eb 205570	Secreted protein
Zm00001eb 205580	Binding
Zm00001eb 205590	Alba DNA/RNA-binding protein
Zm00001eb 205600	Uncharacterized protein
Zm00001eb 205610	Peptidase C13 family; GPI-anchor transamidase

Zm00001eb 205620	Ribosomal RNA-processing protein 8
Zm00001eb 205630	Small ribosomal subunit protein eS8 (40S ribosomal protein S8)
Zm00001eb 205640	Uncharacterized protein
Zm00001eb 205650	Profilin
Zm00001eb 205660	unknown
Zm00001eb 205670	40S ribosomal protein S8
Zm00001eb 205680	unknown
Zm00001eb 205690	Uncharacterized protein
Zm00001eb 205700	unknown
Zm00001eb 205710	40S ribosomal protein S8
Zm00001eb 205720	Protein kinase domain-containing protein
Zm00001eb 205730	Uncharacterized protein
Zm00001eb 205740	FAD-binding PCMH-type domain-containing protein
Zm00001eb 205750	Uncharacterized protein
Zm00001eb 205760	EF-hand domain-containing protein; Calcium-binding mitochondrial carrier F55A11.4
Zm00001eb 205770	DUF4050 domain-containing protein
Zm00001eb 205780	EIN3-binding F-box protein 1; F-box domain-containing protein
Zm00001eb 205790	Seed specific protein Bn15D14A (Seed specific protein1)
Zm00001eb 205800	Uncharacterized protein
Zm00001eb 205810	CRAL/TRIO N-terminal domain-containing protein; CRAL-TRIO domain-containing protein; Sec14p-like phosphatidylinositol transfer family protein
Zm00001eb 205820	26S protease regulatory subunit S10B homolog B
Zm00001eb 205830	F-box domain-containing protein
Zm00001eb 205840	peptidylprolyl isomerase
Zm00001eb 205850	Ribosomal protein L7/L12 C-terminal domain-containing protein
Zm00001eb 205860	DUF868 family protein
Zm00001eb 205870	unknown
Zm00001eb 205880	Uncharacterized protein
Zm00001eb 205890	DNA damage-inducible protein 1; Ubiquitin-like domain-containing protein; Ubiquitin family protein
Zm00001eb 205900	unknown
Zm00001eb 205910	Calcium ion binding protein
Zm00001eb 205920	Regulator of chromosome condensation (RCC1) family protein; Putative regulator of chromosome condensation (RCC1) family protein
Zm00001eb 205930	Fumarylacetoacetase

Table S3 RNA-seq reads of 5 mm immature ear mapped to the maize B73 RefGen_V5 genome

Name	Repeat	Total read	Mapped read	Mapping rate
	Rep-1	43,263,598	40,782,604	0.9427
$qKRN4.09^{BMY}$	Rep-2	44,680,314	42,173,144	0.9439
	Rep-3	45,889,950	43,254,436	0.9426
	Rep-1	47,662,962	45,196,706	0.9483
$qKRN4.09^{B73}$	Rep-2	64,084,852	60,693,264	0.9471
	Rep-3	59,571,998	56,540,998	0.9491

Table S4 Gene-specific primers used in this study

Primer name	Sequences (5'->3')	Usage
Zm00001eb205550-1-F	CCCACCTGTCCTTGAGTCAC	Sequencing
Zm00001eb205550-1-R	CTCAGCACAGCATTGCATCC	
Zm00001eb205550-2-F	TACAAGCCGGAAAGAGGTGC	
Zm00001eb205550-2-R	TTCACCCCACTTCTGCTCAG	
Zm00001eb205550-3-F	ACGTGGACAATGGTGTGTCT	
Zm00001eb205550-3-R	AGGTCGAGCAATTGTGGAGG	
Zm00001eb205550-4-F	TTGCAGGAATGACAGGGTCC	
Zm00001eb205550-4-R	AATGAATACCACGCCCGTCC	
Zm00001eb205550-5-F	ACACAGCCGAGCTTTGCTTA	
Zm00001eb205550-5-R	CGGCGCTGCAGACTTTTTAA	
Zm00001eb205550-qF1	CTGTTGTTTTGTTCTTGCGG	RT-qPCR
Zm00001eb205550-qR1	GCAGCGCTGGCATATGTCTC	
actin-F	TACGAGATGCCTGATGGTCAGGTCA	
actin-R	TGGAGTTGTACGTGGCCTCATGGAC	

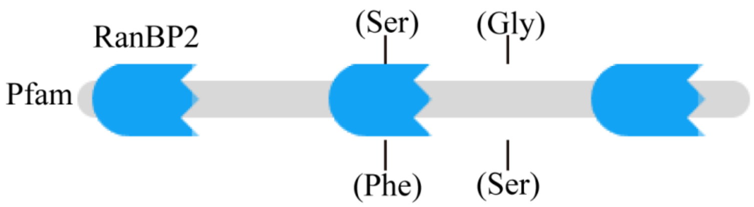

Figure S1 The protein structure of ZmZFP30. The blue column in the Pfam represents the functional domain.
